# Scientific evaluation of the High and Intensive Care (HIC) pilot projects in Belgian mental healthcare

**DOI:** 10.1192/j.eurpsy.2022.1590

**Published:** 2022-09-01

**Authors:** H. Demunter, H. Jossa, K. Vandenhout, S. Claes, R. Bruffaerts

**Affiliations:** 1 UPC Z.org KU Leuven, Hic, Kortenberg, Belgium; 2 KU Leuven, Onderzoeksgroep Psychiatrie, Leuven, Belgium

**Keywords:** High and Intensive Care, coercion, continuity of care

## Abstract

**Introduction:**

The systematic monitoring and evaluation of innovative healthcare programs are essential to develop long-term sustainable solutions that respond to the health needs in the population (Porter & Teisberg, 2006). One such innovative healthcare program is the psychiatric High and Intensive Care (HIC) model, gradually implemented in 9 Belgian psychiatric hospitals since 2019. The HIC-model focuses on intensive patient-oriented care, in an attempt to exclude coercive measures and promote collaborative efforts between staff, patients, and relatives (Voskes et al., 2021).

**Objectives:**

We discuss the following research questions: (1) which clinical profiles of patients are treated in HIC units in Belgium?; (2) Is the implementation of HIC units associated with decrease of coercive measures?; (3) What are self-reported aspects of HIC treatment approaches as experienced by patients, family and/or close friends, and professional staff (both working on the HIC units as well as in external healthcare facilities), and (4) what is the role of HIC units in the organization of mental healthcare on the societal level (e.g. The function of HIC in regional psychiatric networks or the health economic aspects)?

**Methods:**

In order to develop a sustainable policy on HIC in Belgium, we use a scientist-practitioner perspective including a multimethod approach.

**Results:**

The preliminary results of the first six months of data collection will be presented.

**Conclusions:**

The preliminary conclusions of the first six months of data collection will be presented.

**Disclosure:**

No significant relationships.

